# Advances in the Management of Recurrent Cervical Cancer: State of the Art and Future Perspectives

**DOI:** 10.1007/s11912-023-01463-9

**Published:** 2023-10-23

**Authors:** Elena Giudice, Mansoor Raza Mirza, Domenica Lorusso

**Affiliations:** 1 Institute of Obstetrics and Gynecology, Università Cattolica del Sacro Cuore, Rome, Italy; 2 Department of Cancer Treatment, Copenhagen University Hospital, Copenhagen, Denmark; 3 Department of Woman, Child and Public Health, Fondazione Policlinico Universitario “A. Gemelli” IRCCS, Largo Agostino Gemelli 8, 00168 Rome, Italy

**Keywords:** Cervical cancer, Immunotherapy, Immune checkpoint inhibitors, PD-L1; ADC

## Abstract

**Purpose of Review:**

This review aims to give an insight into the currently available options for recurrent/metastatic (R/M) cervical cancer (CC), along with the main future, potentially practice-changing perspectives in this field.

**Recent Findings:**

Improvements in terms of tumor responses were observed with the use of immune checkpoints inhibitors (ICIs) in the previously treated CC population, followed by emerging striking data in terms of both antitumor activity and survival rates with the addition of the ICIs to platinum-based chemotherapy with or without bevacizumab in the first-line setting. Furthermore, the CC treatment landscape took another step forward in 2021 with the introduction of antibody–drug conjugates (ADCs) in the second-line setting, a highly targeted therapeutic strategy, which demonstrated to be a valid alternative option in the recurrent setting.

**Summary:**

R/M CC is a hard-to-treat disease. However, after several years of limited systemic therapeutic options for the recurrent setting, the year 2018 marked a turning point for R/M CC patients, with the introduction of immunotherapy in the treatment paradigm, which completely reshaped the therapeutic armamentarium of the disease. Besides, another valuable treatment option represented by ADCs demonstrated its efficacy in the recurrent setting, thus further widening the treatment landscape for those patients. Yet, the introduction of immunotherapy in the upfront setting brought along new issues to be addressed such as the emerging ICIs resistance and the following need for alternative options in the post-ICIs setting. Several innovative therapeutic strategies are under investigation in ongoing clinical trials, with the aim of overcoming ICIs resistance with the addition of immunomodulatory agents or bypassing the ICIs resistance with novel alternative drugs.

## Introduction

Cervical cancer (CC) is one of the most preventable and treatable forms of cancer, as demonstrated by the 65% reduction in CC incidence rates from 2012 to 2019 in women between the ages of 20 and 24, who were the first to receive the human papillomavirus (HPV) vaccine [1]. However, survival outcomes and incidence rates are highly affected by country-level income and education variables, especially for the HPV-related squamous histotype, underscoring existing global health disparities [2]. The World Health Organization (WHO) launched the Global Cervical Cancer Elimination Initiative to eliminate CC by 2100, but more efforts in terms of screening programs, coverage of HPV vaccination, and expanded access to affordable treatments are required to reach this goal [3]. The importance of the screening in terms of early detection of disease is supported by the evidence of a remarkable difference in survival rates among the different stages of disease: 5-year overall survival (OS) is 92% in the early stages, 65% and 17% in locally advanced and metastatic diseases respectively, with an estimated OS for the recurrent disease of 13 − 17 months [4]. While the standard of care treatment for the early-stage disease remains surgery with curative intent, the treatment paradigms for locally advanced (concomitant chemo-radiotherapy) and recurrent/metastatic (R/M) disease (systemic chemotherapy) have rapidly evolved in recent years. Indeed, despite definitive chemo-radiotherapy, the 5-year disease-free survival (DFS) of patients diagnosed with locally advanced disease (FIGO IB2-IVA) is around 47–80% [5], and the management of women with metastatic (FIGO stage IVB) and recurrent disease has represented an unmet clinical need for decades.

In this context, the advent of immune checkpoint inhibitors (ICIs) has reshaped the treatment paradigm for R/M CC management, showing unprecedented results in different settings. Furthermore, beyond the clinical evidence of antitumor activity and efficacy of ICIs monotherapy in previously treated R/M CC patients, the immunostimulatory properties of chemotherapy agents were leveraged to improve responses in the frontline setting [6], and novel immunomodulatory drugs are currently under investigation to enhance the efficacy of ICIs. Yet, the successful introduction of immunotherapy in the treatment armamentarium for R/M CC patients has raised new issues, such as the choice of treatment suitable for the post-immunotherapy setting, and the immune checkpoint resistance. In this scenario, research is currently focusing on addressing those opening questions, with the development of new treatment strategies, and a more targeted angle.

Antibody drug conjugates (ADCs) represent one of the next frontiers in the paradigm shift towards individualized therapies in CC, as they target specific tumor-associated antigens, with the ability to deliver anticancer drugs directly to the tumor, thus avoiding unnecessary toxicities. Besides, new biomarker-driven immunotherapeutic drugs are being examined, to better select the CC population beyond the programmed death-ligand 1 (PD-L1) status, including cancer therapeutic vaccines targeting E6/E7 oncoproteins for the HPV-related subpopulation, and cell-based strategies targeting specific antigens.

In this review, we aim to provide an overview of the current status of the medical treatment management for R/M CC, along with an updated understanding of future perspectives for this rapidly evolving field.

## Rationale for Immunotherapy in R/M CC

A robust biological rationale supports the use and development of immunotherapeutic strategies to achieve an antitumor activity in CC. Given the nearly universal HPV-associated etiology, the inhibition of the immune system is one of the crucial steps for the development of CC, due to the well-studied mechanisms of immune escape adopted by the HPV virus against immune-mediated clearance [7]. Additionally, high-risk E6/E7 HPV oncoproteins, responsible for the HPV-driven carcinogenesis, can activate the programmed death-1 (PD-1)/PD-L1 axis [8]. Indeed, CC is characterized by high levels of PD-L1, which is positive in up to 90% of CC and is one of the immunological features predictive for immune checkpoint blockade [9]. Based on this evidence, the first studied ICIs in cervical cancer were the PD-1 inhibitors, that prevents the activation of the PD-1/PD-L1 inhibitory signal, by binding the PD-1 receptor on T cells, and thus blocking its interaction with PD-L1 and PD-L2 ligands, therefore preserving T cell proliferation and cytokine production [10].

## The First Step in the CC Immunotherapy Paradigm Shift: ICIs Monotherapy in the Post-platinum Setting

**Fig. 1:**
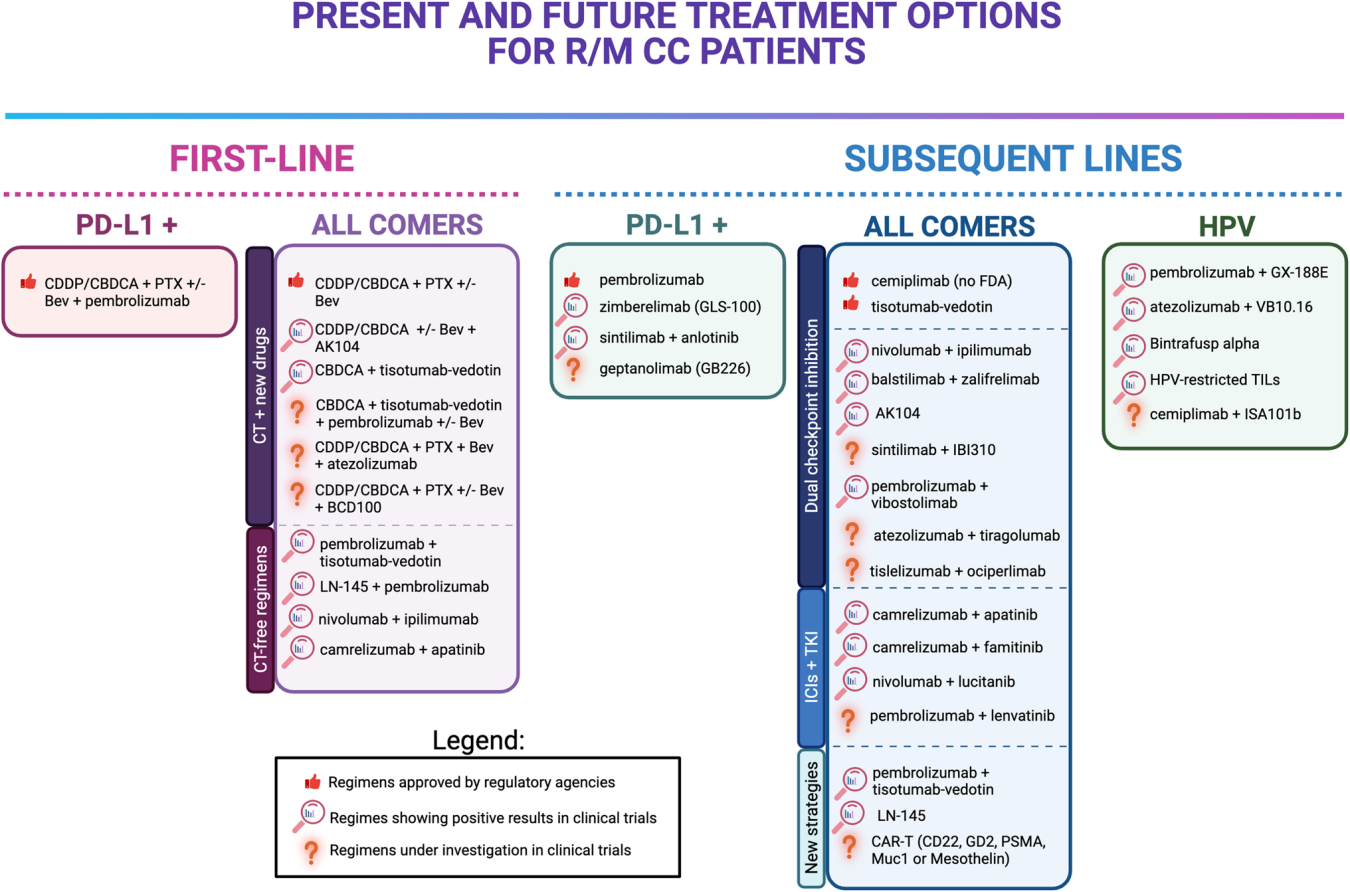
Present and future therapeutic strategies for the treatment of recurrent/metastatic cervical cancer (R/M CC). Programabsmed death-ligand 1 (PD-L1); cisplatin (CDDP); Carboplatin (CBDCA); Paclitaxel (PTX); Bevacizumab (Bev); human papillomavirus (HPV); tumor-infiltrating lymphocytes (TILs); chimeric antigen receptor (CAR); cluster of differentiation-22 (CD22); prostate-specific membrane antigen (PSMA); Mucin 1 (MUC1)

Metastatic and recurrent CC is traditionally considered an incurable disease, requiring palliative chemotherapy. A significant step forward in defining the optimal therapy for this setting was done in 2009 when the platinum-taxane doublet therapy has been proven to be the backbone treatment for CC, albeit the still disappointing response rate (RR) of 36% and median overall survival (mOS) of 12.87 months (95% confidence interval [CI], 10.02 to 16.76 months) [11]. A few years later, given the evidence of HPV-mediated tumor angiogenesis, through the increasing levels of HIF-1α protein, and the upregulation of vascular endothelial growth factor (VEGF) [12], the anti-VEGF monoclonal antibody, bevacizumab, was tested in combination with platinum-based chemotherapy [13], demonstrating increased overall survival (OS: 17.0 months vs. 13.3 months; hazard ratio [HR] 0.71; 98% CI: 0.54–0.95; *p* = 0.004) and higher RR (48% vs. 36%, *P* = 0.008). Final OS at 20.8 months of follow-up published in 2017 [14], confirmed the clinically meaningful and statistically significant survival benefit among CC patients treated with bevacizumab in addition to standard platinum-based chemotherapy. After almost three decades of poor OS data, this trial demonstrated for the first time a gain in survival rates, thus setting a new milestone in the CC treatment paradigm. After the failure of the first-line treatment, available chemotherapy options have demonstrated limited clinical activity, with objective response rate (ORR) ranging from 4.5 to 13.7% [15]. Until 2021, no other valuable alternative options were found. In this scenario, given the lack of universally accepted standard of care therapies, the development of an alternative strategy was urgently needed. The robust rationale behind the use of immune checkpoint blockade to enhance the anticancer immune response prompted researchers to investigate the effect of ICIs in this setting (Table 1). Impressive results from clinical trials have unleashed the first step towards a paradigm shift in CC therapeutic management (Fig. 1).

**Table 1:** Efficacy data from clinical trials for previously treated R/M CC patients

	NCT	Study name	Phase	Drugs	Targets	ORR	DoR	PFS	OS	References
Clinical Trials leading to FDA and/or EMA drug approvals										
	NCT02054806	KEYNOTE-028	Ib	Pembrolizumab	PD-1	ORR: 17% (95% CI, 5 to 37)	mDoR: 5.4mo (95% CI, 4.1 to 7.5)	mPFS: 2.5mo	mOS: 11mo	Frenel et al., 2017
	NCT02628067	KEYNOTE-158	II	Pembrolizumab	PD-1	ORR: 14.3% (95% CI, 8 to 22.8)	mDoR: NR (95% CI, 3.7 to 35.2)	mPFS:2.1mo (95% CI, 2.0 to 2.2 months	mOS: 9.4mo (95% CI, 7.7 to 13.1)	Chung et al., 2020
	NCT02001623	InnovaTV 201	I/II	Tisotumab-vedotin	TF/MMAE	ORR: 15.6% (95% CI 10.2 to 22.5)	5.7mo (95% CI 3.0 to 9.5)			de Bono et al., 2019
	NCT03913741	InnovaTV 206	I/II	Tisotumab-vedotin	TF/MMAE	ORR: 29.4% (95% CI, 10.3–56.0)	7.1mo (3.1 to NR)	mPFS: 3.1mo (95% CI, 1.2 to 7.1)	mOS: 11.4mo (95% CI, 6.2 to NR)	Yonemori et al., 2022
	NCT03438396	InnovaTV 204//GOG-3023/ENGOT-cx6	II	Tisotumab-vedotin	TF/MMAE	ORR: 24% (95% CI 16 to 33)	8.3mo (95% CI 4.2 to NR)	mPFS: 4.2mo (95% CI 3.0 to 4.4)	mOS:12.1mo (95% CI 9.6 to 13.9)	Coleman et al., 2021
	NCT03257267	EMPOWER-Cervical 1/GOG-3016/ENGOT-cx9	III	Cemiplimab	PD-1	ORR: 16.4% (95% CI, 12.5 to 21.1)	mDoR: 16.4mo (95% CI, 12.4 to NR)	mPFS: 2.8mo (95% CI, 2.6 to 3.9)	mOS: 12mo (vs. chemo mOS: 8.5mo, HR 0.69 (95% CI, 0 0.56 to 0.84)	Tewari et al., 2022
Alternative ICIs monotherapy in the PD-L1-positive population										
	NCT03972722		II	Zimberelimab	PD-1	ORR: 27.8% (95% CI, 18.85 to 38.22)	6-mo DoR: 84% (95% CI, 58 to 95)	mPFS: 3.7mo	12-mo OS: 54% (95% CI, 41 to 66)	Wu et al., 2022
	ChiCTR1900023015		II	Sintilimab + Anlotinib	PD-1 + TKI	ORR: 54.8% (95% CI, 38.7 to 70.2)		mPFS: 9.4 months (95% CI, 8.0 to 14.6)	mOS: NR (95% CI, 12.3 to NR)	Xu et al., 2022
ICIs monotherapy in the overall population										
	NCT02257528	NRG-GY002	II	Nivolumab	PD-1	ORR: 4% (90% CI, 0.4% to 22.9%)	mDoR: 3.8mo	mPFS: 3.5mo (90% CI: 1.9 to 5.1)	mOS: 14.5mo (90% CI: 8.3 to 26.8)	Santin et al., 2020
	NCT03104699		II	Balstilimab	PD-1	ORR: 15% (95% CI, 10.0%-21.8%)	mDoR: 15.4mo (95% CI, 5.7mo to NR)			O’Malley et al., 2021
	NCT01693783		I/II	Ipilimumab	CTLA-4			mPFS: 2.5mo (95% CI, 2.1 to 3.2)	mOS: 8.5mo (95% CI, 3.6 to NR)	Lheureux et al., 2018
Dual checkpoint inhibition										
	NCT02488759	CheckMate 358	I/II	Nivolumab Nivolumab + Ipilimumab (N3 + I1) Nivolumab + Ipilimumab (N1 + I3)	PD-1 PD-1 + CTLA-4 PD-1 + CTLA-4	ORR: 26% (95% CI 9 to 51) ORR: 26% (95% CI 11 to 46) ORR: 35% (95% CI 21 to 51)	mDoR: NR (35.3 to NR) mDoR: 21.1mo (95% CI, 7.5 to NR) mDoR: NR (95% CI, 5.2 to NR)	mPFS: 5.1mo (95% CI 1.9 to 9.1) mPFS: 3.8mo (95% CI 2.1 to 10.3) mPFS: 5.8mo (95% CI 3.8 to 9.3)	mOS: 21.6mo (95% CI 8.3 to 46.9) mOS: 15.2mo (95% CI 9.0 to 36.2) mOS: 20.9mo (95% CI 14.4 to 2.8)	Oaknin et al., 2022
	NCT03495882		II	Balstilimab + Zalifrelimab	PD1 + CTLA-4	ORR: 25.6% (95% CI, 18.8 to 33.9)	mDoR: NR (95% CI, 9.7 to NR)	mPFS: 2.7 mo (95% CI, 1.5 to 3.7	mOS: 12.8mo (95% CI, 8.8 to 17.6)	O’Malley et al., 2022
	NCT03852251		II	AK104	PD-1/CTLA-4	ORR: 33% (95% CI, 23.9 to 43.1)	mDoR: NR (95% CI, 0.95 to 16.43)	mPFS: 3.75mo (95% CI, 0.03 to 18.46)	mOS: 17.5mo (95% CI, 0.62 to 19.78)	Xiaohua et al., 2022
	NCT02964013	KEYVIBE-001	I	Pembrolizumab + vibostolimab 200 mg Pembrolizumab + vibostolimab 700 mg	PD-1 + TIGIT	ORR: 15% ORR: 23%	mDoR: NR (10 to 31 +) mDoR: NR (4 + to 35 +)	mPFS: 2mo (95% CI, 2 to 4) mPFS: 2mo (95% CI, 2 to 4)		Shapira-Frommer et al., 2022
ICIs in combination TKI										
	NCT03816553	CLAP	II	Camrelizumab + Apatinib	PD-1 + VEGFR2	ORR: 55.6% (95% CI, 40.0% to 70.4%)	mDoR: NR (NR (95% CI, 5.6 to NR)	mPFS: 8.8mo (95% CI, 5.6 to NR)	mOS: NR (95% CI, 11.6 to NR)	Lan et al., 2020
	NCT03827837		II	Camrelizumab + Famitinib	PD-1 + TKI	ORR: 39.4% (95% CI, 22.9 to 57.9)	mDoR: NR (95% CI, 8.2 to NR)	mPFS: 10.3mo (95% CI, 3.5 to NR)	12-mo OS: 77.7% (95% CI: 58.9–88.7)	Xia et al., 2022
	NCT04042116	LIO-1	II	Nivolumab + Lucitanib	PD-1 + TKI	ORR: 31.8%		mPFS: 5.5mo (95% CI, 3.2 to 10.9)		Patel et al., 2022
Alternative drugs in combination with ICIs										
	NCT03786081	ENGOT Cx8/GOG 3024/innovaTV-205	I/II	Pembrolizumab + Tisotumab-vedotin	PD-1 + TF/MMAE	ORR: 35% (95% CI, 20 to 54)		mPFS: 5.6mo (95% CI 2.7 to 9.6)		Vergote et al., 2021
	NCT03444376		II	Pembrolizumab + GX-188E	PD-1 + E6/E7 DNA vaccine	ORR: 31%				Lee et al., 2022
	NCT04405349	VB C-02	II	Atezolizumab + VB10.16	PD-L1 + E6/E7 DNA vaccine	ORR: 21%				Nykode Therapeutics, 2022
	NCT03439085		II	Durvalumab + MEDI0457	PD-L1 + E6/E7 DNA vaccine	ORR: 0%				Morris et al., 2021
	NCT02517398, NCT03427411		I,II	Bintrafusp alpha	TGF-β/PD-L1	ORR: 28.2%	mDoR: 11.7mo (1.4 to 41.2)		mOS: 13.4mo (95% CI, 5.5 to NR)	Strauss et al., 2021
Innovative drugs										
	NCT03108495	C-145–04/innovaTIL-04	II	LN-145	TILs	ORR: 44%				Jazaeri et al., 2019
	NCT02280811	I/II		ACT	TCR-E6 restricted TILs	ORR: 0%				Doran et al., 2019
	NCT01585428		II	ACT	HPV-restricted TILs	ORR: 28%				Stevanović S et al., 2019

*ORR* objective response rate, *DoR* duration of response, *PFS* progression-free survival, *OS* overall survival, *FDA* US Food and Drug Administration, *EMA* European Medicines Agency, *PD-1* programmed death-1, *CI* confidence interval, *mDoR* median duration of response, *mo* months, *mPFS* median progression-free survival, *mOS* median overall survival, *TF* tissue factor, *MMAE* monomethyl auristatin E, *NR* not reached, *HR* hazard ratio, *PD-L1* programmed death-ligand 1, *TKI* tyrosine kinase inhibitors, *CTLA-4* cytotoxic T lymphocyte-associated protein 4, *N3* + *I1* nivolumab 3 mg/kg every 2 weeks + ipilimumab 1 mg/kg every 6 weeks, *N1* + *I3* nivolumab 1 mg/kg every 2 weeks + ipilimumab 3 mg/kg every 3 weeks × 4 cycles followed by nivolumab 240 mg every 2 weeks (N1 + I3), *TIGIT* T cell immunoreceptor with immunoglobulin and ITIM domains, *VEGFR2* vascular endothelial growth factor receptor 2, *TGF-β* transforming growth factor-beta, *TILs* tumor-infiltrating lymphocytes, *ACT* adaptive T cell therapy, *TCR* T cell receptor, *HPV* human papillomavirus

In the phase Ib KEYNOTE 028 trial, pembrolizumab demonstrated meaningful clinical activity in patients with R/M CC for the first time [16•], showing an ORR of 17% and a median duration of response (mDoR) of 5.4 months in tumors expressing PD-L1. The phase II KEYNOTE 158 trial, enrolling patients regardless of PD-L1 status, further confirmed the antitumor activity only in the PD-L1 subgroup, with an ORR of 17.1% (95% CI 9.7 to 27%) in the PD-L1 positive subgroup, and an ORR of 14.3%, 95% CI 8 to 22.8%) in the overall CC cohort (both PD-L1 positive and negative tumors) [17••]. Based on these results, FDA granted the accelerated approval of pembrolizumab as second-line treatment for CC with combined positive score (CPS) ≥ 1 in June 2018, and marked a turning point in the management of CC, with the introduction of immunotherapy in the CC treatment armamentarium (Fig. 1). The impressive clinical activity of pembrolizumab in the PD-L1-positive R/M CC yielded the spread of clinical trials exploring the safety and efficacy of new anti-PD-1 agents for this biomarker-selected population. Geptanolimab (GB226) and zimberelimab (GLS-100) are two PD-1 inhibitors that have been evaluated for the treatment of PD-L1-positive, R/M CC after the failure of the first-line treatment, alone or in combination with other agents. Zimberelimab monotherapy was tested in a single arm, phase II study, showing promising prolonged tumor responses (6-month DoR rate: 84%, 95% CI, 58 to 95%), clinically meaningful antitumor activity, significantly higher compared to historical controls (ORR: 27.8%, 95% CI: 18.85 to 38.22) and acceptable toxicity in PD-L1 positive, CC patients in the post-platinum setting, after a median follow-up of 11.5 months (Table 1) [18]. GB226 is another highly selective PD-1 inhibitor under investigation in an open-label, single-arm, phase II clinical trial, whose estimated primary endpoint completion was expected in December 2021; however, no results have been already posted (NCT03808857, Table 2).

**Table 2:** Ongoing clinical trials investigating new treatments for R/M CC patients

	NCT	Study name	Phase	Drugs	Targets	Primary endpoint	Status
First-line							
	NCT03556839	BEATcc (ENGOT-Cx10/GEICO 68-C/JGOG1084/GOG-3030)	III	CDDP/CBDCA + PTX + Bev ± atezolizumab	CT + VEFG-A ± PD-L1	PFS, OS	Active, not recruiting
	NCT03912415	FERMATA	III	CDDP/CBDCA + PTX ± Bev ± BCD100	CT ± VEFG-A ± PD-1	OS	Recruiting
	NCT03786081	ENGOT Cx8/GOG 3024/innovaTV 205	I/II	CBDCA ± Bev + pembrolizumab + TV	CT ± VEFG-A + PD-1 + TF/MMAE	ORR	Active, not recruiting
	NCT04982237		III	CDDP/CBDCA + PTX ± Bev ± AK104	CT ± VEFG-A + PD-1/CTLA-4	PFS, OS	Recruiting
	NCT04974944		II	Camrelizumab + Apatinib vs. CDDP/CBDCA + PTX + Bev	PD-1 + VEGFR2 vs. CT + VEGF-A	PFS	Recruiting
Second (or later) lines							
	NCT04697628	ENGOT CX12/GOG-3057/INNOVATV 301	III	TV vs. standard of care CT	TF/MMAE vs. CT	OS	Recruiting
	NCT03808857		II	Geptanolimab (GB226)	PD-1	ORR	UNKNOWN
	NCT03894215	RaPiDS/GOG-3028	II	Balstilimab ± Zalifrelimab	PD-1 ± CTLA-4	ORR	Active, not recruiting
	NCT04590599		II	Sintilimab + IBI310	PD-1 ± CTLA-4	ORR	Recruiting
	NCT04693234	AdvanTIG-202	II	Tislelizumab + Ociperlimab (BGB-A1217)	PD-1 + TIGIT	ORR	Active, not recruiting
	NCT05007106		II	Pembrolizumab ± vibostolimab	PD-1 + TIGIT	ORR	Recruiting
	NCT04300647	SKYSCRAPER-04	II	Atezolizumab ± Tiragolumab	PD-1 + TIGIT	ORR	Active, not recruiting
	NCT05007106		II	Pembrolizumab ± vibostolimab	PD-1 + TIGIT	ORR	Recruiting
	NCT04680988		II	Camrelizumab ± famitinib vs. standard of care chemotherapy	PD-1 + TKI vs. CT	ORR	Active, not recruiting
	NCT04865887		II	Pembrolizumab and Lenvatinib	PD-1 + TKI	ORR	Recruiting
	NCT04646005		II	Cemiplimab + ISA101b	PD-1 + E6/E7 peptide-based vaccine	ORR	Active, not recruiting
	NCT04556669		I	CAR-TILs	CD22	SAFETY	Recruiting
	NCT03356795		I/II	CAR-TILs	GD2, PSMA, Muc1 or Mesothelin	SAFETY	UNKNOWN

*CDDP* cisplatin, *CBDCA* carboplatin, *PTX* paclitaxel, *Bev* bevacizumab, *CT* chemotherapy, *VEFG-A* vascular endothelial growth factor-alpha, *PD-L1* programmed death-ligand 1, *PFS* progression-free survival, *OS* overall survival, *PD-1* programmed death-1, *TV* tisotumab-vedotin, *TF* tissue factor, *MMAE* monomethyl auristatin E, *ORR* objective response rate, *CTLA-4* cytotoxic T lymphocyte-associated protein 4, *VEGFR2* vascular endothelial growth factor receptor 2, T cell immunoreceptor with immunoglobulin and ITIM domains, *TKI* tyrosine kinase inhibitors, *CAR* chimeric antigen receptor, *TILs* tumor-infiltrating lymphocytes, *CD22* cluster of differentiation-22, *PSMA* prostate-specific membrane antigen, *MUC1* Mucin 1

However, given the restricted indication of pembrolizumab only for CC patients with PDL1-positive tumors, alternative strategies have been investigated to extend the indication of ICIs to a broader CC population. In this context, other ICIs monotherapy has been studied. Yet, other PD-1 inhibitors (nivolumab, bastilimab), and the cytotoxic T lymphocyte-associated protein 4 (CTLA-4) inhibitor ipilimumab showed only limited activity in clinical trials investigating their activity as single agents in previously treated CC patients (Table 1) [19–21]. Following those results, those agents have been further investigated in clinical trials to test synergistic combinations to increase their clinical responses in previously treated CC patients. In contrast, positive results came from the randomized, phase III, EMPOWER-Cervical 1/GOG-3016/ENGOT-cx9, investigating the anti-PD-1 antibody cemiplimab as a single agent compared with standard chemotherapy in previously treated, R/M CC patients. Cemiplimab showed promising clinical activity (ORR: 16.4%, 95% CI: 12.5 to 21.1), and durable responses (mDoR: 16.4 months, 95% CI: 12.4 to not reached [NR]), along with a higher mOS compared with chemotherapy (mOS of 12 months vs. 8.5 months; HR 0.69, 95% CI: 0 0.56 to 0.84), thus leading to early termination of the trial due to significant benefit. Even more important, cemiplimab displays its antitumor activity regardless of PD-L1 status, tumor histology, performance status, and the number of prior lines, thus demonstrating a broadened activity compared to pembrolizumab (Table 1) [22••]. Yet just a month after the FDA license application, the sponsor decided to withdraw it due to a misalignment between the sponsor and FDA concerning post-marketing studies. On the contrary, other regulatory agencies around the world, including the European Commission, granted its approval irrespective of the PD-L1 status as second-line treatment (Fig. 1).

## Bringing Immunotherapy Forefront: ICIs in Combination with Chemotherapy

The evidence of the immunomodulatory activity of platinum-based chemotherapy, along with preclinical data suggesting extensive crosstalk between the angiogenic pathway and the anti-tumor immune response have set the foundation to investigate the standard of care platinum-based chemotherapy plus bevacizumab, in combination with the ICIs in the clinical setting [23]. The use of the programmed death-1 (PD-1) inhibitor pembrolizumab, in combination with platinum-based agents with or without bevacizumab, demonstrated to be a clinically meaningful treatment strategy to prolong survival rates for chemo naïve R/M CC patients, according to remarkable progression-free survival (PFS) and OS data reported in the practice-changing, randomized phase III, KEYNOTE-826 trial (Table 3) [24••]. Specifically, an unprecedent ORR of 65.9% (95% CI 60.3 to 71.2) was observed with the addition of pembrolizumab to the standard platinum-based chemotherapy, with a mDoR of 18.0 months. Besides, median PFS (mPFS) was significantly improved in the experimental arm, compared to the standard treatment (10.4 vs. 8.2 months; HR 0.65, 0.53 to 0.79, *p* < 0.001) and mOS as well (24.4 vs. 16.5 months; HR 0.67, 0.54 to 0.84, *p* < 0.001). Despite a clear clinical benefit and the statistical significance across all the subgroups both in terms of PFS and OS shown in the KEYNOTE826 study, pembrolizumab in combination with the standard of care first-line treatment (platinum-based chemotherapy ± bevacizumab) granted the regulatory agency approvals by the US Food and Drug Administration (FDA) and European Medicines Agency (EMA) exclusively for patients with a significant tumor expression of PD-L1, defined as CPS ≥ 1, thus warranting further investigation.

**Table 3:** Efficacy data from clinical trials for chemo naïve, R/M CC patients

	NCT	Study name	Phase	Drugs	Targets	ORR	DoR	PFS	OS	References
Clinical Trials leading to FDA and/or EMA drug approvals										
	NCT03635567	KEYNOTE826	III	CDDP/CBDCA + PTX ± Bev ± pembrolizumab	CT ± VEFG-A ± PD-1	ORR: 65.9% (95% CI 60.3 to 71.2)	mDoR: 18.0mo vs. 10.4mo	mPFS: 10.4mo vs 8.2mo (HR 0.65, 0.53 to 0.79, *p* < 0.001)	mOS: 24.4mo vs 16.5mo (HR 0.67, 0.54 to 0.84, *p* < 0.001)	Colombo et al., 2021
Alternative treatment options in combination with chemotherapy										
	NCT04864782		II/III	CDDP/CBDCA + PTX ± QL1604	CT ± PD-1	ORR 58.7% iORR 60.9%	mDOR: 9.6mo (95% CI, 5.5 to NE)	mPFS: 8.1mo (95% CI, 5.7 to NE)		Liu J et al., 2022
	NCT03786081	ENGOT-cx8/GOG-3024/innovaTV 205	I/II	CBDCA + TV	CT + TF/MMAE	ORR: 55% (95% CI 36 to 72)	mDOR: 5.6mo (95% CI 2.7 to NE)	mPFS: 6.9mo (95% CI 3.9 to NE)		Vergote et al., 2021
	NCT04868708		II	CDDP/ CBDCA + AK104 15 mg/kg CDDP/ CBDCA + AK104 10 mg/kg CDDP/ CBDCA + Bev + AK104 10 mg/kg	CT ± VEFG-A ± PD-1/CTLA-4	ORR:73.3% ORR: 68.8% ORR: 92.3%	mDOR: NR (95% CI, 2.99 to NE)	mPFS: 5.75mo (95% CI, 2.86 to NE)		Wang et al., 2022
	ChiCTR1800018585		II	CDDP/CBDCA + PTX + Endostar	CT + KDR/VEGFR2	ORR: 50%		mPFS: 12mo (95%CI, 8.33 to 15.67		Zhou et al., 2021
Alternative treatment options replacing chemotherapy										
	NCT02488759	CheckMate 358	I/II	Nivolumab Nivolumab + Ipilimumab (N3 + I1) Nivolumab + Ipilimumab (N1 + I3)	PD-1 PD-1 + CTLA-4 PD-1 + CTLA-4	ORR: 26% (95% CI 9 to 51) ORR: 39% (95% CI 17 to 64) ORR: 41% (95% CI 29 to 53)	NR (95% CI, 35.3 to NR) 34.6mo (95% CI, 6.6 to NR) 35.6mo (95% CI, 9.2 to NR)	mPFS: 5.1mo (95% CI 1.9 to 9.1) mPFS: 3.8mo (95% CI 2.1 to 10.3) mPFS: 5.8mo (95% CI 3.8 to 9.3)	mOS: 21.6mo (95% CI 8.3 to 46.9) mOS: 15.2mo (95% CI 9.0 to 36.2) mOS: 20.9mo (95% CI 14.4 to 2.8)	Oaknin et al., 2022
	NCT03786081	ENGOT Cx8/GOG 3024/innovaTV-205	I/II	Pembrolizumab + TV	PD-1 + TF/MMAE	ORR: 41% (95% CI 24 to 59)	NR	mPFS: 5.3 mo (95% CI, 4.0 to 12.2)		Lorusso et al., 2022
	NCT03108495	C-145–04/innovaTIL-04	II	LN-145 TILs + pembrolizumab	TILs + PD-1	ORR: 57% (95% CI, 28.9, 82.3)				O’Malley et al., 2021

*ORR* objective response rate, *DoR* duration of response, *PFS* progression-free survival, *OS* overall survival, *FDA* US Food and Drug Administration, *EMA* European Medicines Agency, *CDDP* cisplatin, *CBDCA* carboplatin, *PTX* paclitaxel, *Bev* bevacizumab, *CT* chemotherapy, *VEFG-A* vascular endothelial growth factor-alpha, *PD-1* programmed death-1, *CI* confidence interval, *mo* months, *mDoR* median duration of response, *mPFS* median progression-free survival, *HR* hazard ratio, *mOS* median overall survival, *iORR* ORR per iRECIST, *NE* not estimable, *TV* tisotumab-vedotin, *TF* tissue factor, *EMMAE* monomethyl auristatin E, CTLA-4 cytotoxic T lymphocyte-associated protein 4, *NR* not reached, *KDR/VEGFR2* kinase insert domain receptor/vascular endothelial growth factor receptor 2, nivolumab 3 mg/kg every 2 weeks + ipilimumab 1 mg/kg every 6 weeks (N3 + I1); nivolumab 1 mg/kg every 2 weeks + ipilimumab 3 mg/kg every 3 weeks × 4 cycles followed by nivolumab 240 mg every 2 weeks (N1 + I3); tumor-infiltrating lymphocytes (TILs)

Following the FDA approval of pembrolizumab combined with platinum-based chemotherapy with or without bevacizumab as first-line treatment for R/M CC expressing PD-L1 (CPS ≥ 1), the subsequent knock-on effect was the spread of similar clinical trials currently investigating ICIs in the same setting (Table 2). The BEATcc study (ENGOT-Cx10/GEICO 68-C/JGOG1084/GOG-3030, NCT03556839) is a multicenter, randomized phase III trial investigating the efficacy of atezolizumab (anti-PD-L1) in combination with platinum-based chemotherapy plus bevacizumab, whose recruitment was completed in September 2021 and final data collection for primary outcome measures (PFS, OS) estimated for completion in March 2023 [25]. Besides, the phase III FERMATA study (NCT03912415) is currently ongoing to evaluate the efficacy of an alternative PD-1 inhibitor, BCD-100, in combination with platinum-based chemotherapy with or without bevacizumab in the same setting, but the enrollment is restricted only to R/M CC patients with squamous histology. OS is the primary endpoint, estimated to be completed by December 2024 (Table 2). Consistent results with KEYNOTE-826 data were recently reported from a Chinese single-arm, open-label phase II study of QL1604 (anti-PD-1) in combination with chemotherapy in patients with R/M CC. In this trial, after a median follow-up was 12.91 months, ORR was 58.7%, mDoR was 9.6 months, and PFS was 8.1 months (Table 3) [26].

## The Post-ICIs Setting: Calling for New Therapeutic Approaches and Algorithms

Multiple compounds are currently under investigation at different stages of development to fill the remaining gaps in the first and the second-line (or later) of treatment in the rapidly evolving treatment paradigm for R/M CC. Building on the success of pembrolizumab in the first-line setting, the identification of alternative therapeutic options in the subsequent lines became a new clinical unmet need. In the precision oncology era, tailored treatments are developed with the purpose of increasing the efficacy of anticancer drugs by minimizing at the same time their systemic distribution and targeting specific molecular markers, selectively expressed by cancer cells, thus avoiding off-target effects. In this context, ADCs represent a new promising strategy to address this issue. Additionally, ICIs combination therapies, including dual checkpoint inhibition and multi-drug approaches to complement ICIs using TME-immunomodulating agents, are currently being evaluated to address the emerging immune checkpoint blockade resistance issue, which will be the next unmet clinical need arising from the current extensive use of immunotherapy in earlier settings.

Moving forward, since different trials are currently investigating the effect of immunotherapy in addition to chemoradiation in the management of locally advanced CC, ICIs may move into earlier stages of therapy. Hence, there might be the need for novel approaches in the next future even in the upfront setting. In this scenario, ICIs combination treatments may represent a promising approach to replace chemotherapy or as an alternative option in patients who do not tolerate it. Besides, alternative agents to ICIs in the first-line setting are under investigation in several clinical trials to enhance the efficacy of chemotherapy.

### ADCs: Effective Agents Alternative to ICIs in the Post-platinum Setting and Promising Drugs to Complement ICIs and Chemotherapy

With their complex structure, composed of three main components, ADCs target specific antigens expressed by tumor surfaces through the interaction of the monoclonal antibody (mAb) with the specific target, which covalently binds a cytotoxic agent via a chemical linker. Moreover, beyond the cytotoxic effect on the targeted cell, driven by the release of the payload in the lysosomes with the consequent cell apoptosis or death via targeting DNA or microtubules, ADCs are able to provoke a bystander effect on neighborhoods cells, and to alter the tumor microenvironment (TME), further enhancing their antitumor effect [27]. The evidence of a high expression of tissue factor (TF) on CC surfaces, along with its critical role in promoting tumor growth and progression, and angiogenesis within the TME [28], led to the development of a new ADC, tisotumab-vedotin (TV), which binds TF through a fully humanized mAb linked via a cleavable mc-VC-PABC to the cytotoxic payload monomethyl auristatin E (MMAE), an antimitotic agent which induces G2/M cell cycle arrest, finally leading to cell death [29]. Adverse events (AEs) of special interest (AESI) are manageable, represented by ocular toxicity, neuropathy, and bleeding. Conjunctivitis (26%), dry eye (23%), and keratitis (11%) are the most common treatment-related ocular events, the majority of those managed with corticosteroids, vasoconstrictors eye drops, and recovered within 30 days after the last dose. Besides, Grade < 3 bleedings frequently occur, including the most common epistaxis (30%), followed by vaginal hemorrhage (7%), and hematuria (3%), more likely without any intervention required. Finally, neuropathy (peripheral, sensory, or sensorimotor) is generally short-term (time to resolution: 0.6 months) and Grade < 3 [30•].

#### ADCs Monotherapy

The first-in-human, phase I/II, innovaTV 201 study showed promising antitumor activity of TV among patients with refractory solid tumors (ORR: 15.6%, 95% CI 10.2 to 22.5%; mDoR: 5.7 months, 95% CI 3.0 to 9.5 months) [30•], and encouraging results were observed in the previously treated, CC cohort (ORR: 24%, mDoR: 4.2 months, and 6-month PFS: 29%) [31]. ORR of 24% was further confirmed in the phase II InnovaTV 204//GOG-3023/ENGOT-cx6, specifically designed to investigate the antitumor activity in pre-treated, R/M CC patients, demonstrating even better results in terms of mDoR (8.3 months, 95% CI 4.2 to NR) and mPFS (4.2 months, 95% CI 3.0 to 4.4 months) [32••]. Similar results were also observed in the InnovaTV 206 study, showing an ORR of 29.4% (95% CI, 10.3% to 56.0%), mDoR of 7.1 months (3.1 months to NR), mPFS of 3.1 months (95% CI, 1.2 to 7.1 months), and mOS of 11.4 months (95% CI, 6.2 months to NR) in a Chinese patient population [33] (Table 1). These data led to the first FDA-approval of ADCs in the CC treatment landscape in late 2021, giving a valid alternative therapeutic option to pembrolizumab, especially for PD-L1-positive, CC patients previously treated in the first-line setting with pembrolizumab in addition to platinum-based chemotherapy with or without bevacizumab (Fig. 1). The randomized, phase III, ENGOT CX12/GOG-3057/INNOVATV 301 trial is ongoing to finally confirm the efficacy of TV compared to standard chemotherapy in previously treated R/M CC patients (NCT04697628, Table 2).

#### ADCs: Combination Approaches

Given the immunomodulatory properties of ADCs and the effectiveness of TV in CC patients regardless the PD-L1 status, this novel therapeutic approach is under investigation in several clinical trials in combination with other anticancer-agents (Fig. 1).

In the first-line setting, TV demonstrated to be a valid alternative option to ICIs in order to complement chemotherapy. The second part of the phase Ib/II ENGOT-cx8/GOG-3024/innovaTV 205 clinical trial demonstrated promising antitumor activity with acceptable safety profile with the use of TV in combination with carboplatin in chemo naïve, R/M CC patients. Preliminary results showed an ORR of 55% (95% CI 36 to 72), which is higher than the historical RR (48%) observed with platinum-based chemotherapy given in combination with bevacizumab [14]. Besides, a mDoR of 5.6 months, and mPFS of 6.9 months at a median follow-up of 4.8 months were observed (Table 3), along with manageable toxicity (ocular events grade 1–2 and grade ≥ 3 in 55% and 3% of patients respectively), peripheral neuropathy (grade 1–2: 27%, grade ≥ 3: 12%) and bleeding events (grade 1–2: 48%, grade ≥ 3: 6%). [34]. Further, the above-cited immunogenic properties of TV, along with the proven ADCs ability to increase T lymphocyte infiltration and to induce dendritic cell maturation, and co-stimulatory molecules expression, gave the basis to explore the synergistic effect of TV in combination with immunotherapy [35, 36]. Indeed, the combination of TV and ICIs was tested both in the chemo-naïve setting and after the failure of the first-line treatment in different trials (Tables 1 and 3).

In the first-line setting, the combination of TV with pembrolizumab was investigated with the aim of replacing chemotherapy, showing an ORR of 41% (95% CI 24 to 59) and mPFS of 5.3 months (95% CI, 4.0 to 12.2) in a cohort of the ENGOT Cx8/GOG 3024/innovaTV-205 trial, thus confirming the synergistic activity of TV and pembrolizumab in the chemo naïve CC population, with manageable toxicity (alopecia: 61%, diarrhea: 55%, epistaxis: 49%, conjunctivitis: 46%, and nausea: 46%) [37]. Besides, TV is currently under investigation in a cohort of the phase Ib/II ENGOT-cx8/GOG-3024/innovaTV 205, even to test the addition of this agent to the quadruplet therapy studied in the KEYNOTE-826 study, which might be a promising strategy to complement chemotherapy in the first-line setting for R/M CC patients who are not eligible to pembrolizumab plus platinum-based chemotherapy because of CPS < 1 (Table 2) [38].

Finally, TV-pembrolizumab combination therapy was also investigated in the recurrent setting after the failure of the first-line treatment. Clinical benefit was observed in the previously treated cohort of R/M CC patients enrolled in the phase I/II ENGOT Cx8/GOG 3024/innovaTV-205 trial. Indeed, promising ORR was observed (ORR: 38%, 95% CI: 22 to 56), along with durable responses (mDoR: 13.8 months, 95% CI: 2.8 to NR), and encouraging survival rates (mPFS: 5.6, 95% CI: 2.7 to 13.7 months; mOS: NR, range 1.3–17.5 + months) after median 13 months of follow-up (Table 1) [34].

### Combination Strategies to Overcome ICI Resistance: Dual Checkpoint Inhibition and Immunomodulatory Agents in Addition to ICIs

A promising approach deeply investigated in the clinical setting to overcome resistance and broaden the efficacy of anti-PD-1 agents is the concomitant dual checkpoint inhibition, by concomitantly blocking two co-inhibitory pathways. The most reliable targets to achieve a synergistic effect with anti-PD-1 antibodies include the blockade of CTLA-4 and T cell immunoreceptor with immunoglobulin and ITIM domains (TIGIT) co-inhibitory signals. CTLA-4 and PD-1 are potent immune checkpoint cell surface receptors involved in the downregulation of T cell response to physiologically establish cell tolerance. However, their expression patterns are spatially and temporally distinct [39], thus suggesting a complementary activity of those two pathways in limiting autoreactivity. Based on this rationale, the dual inhibition of those pathways entered the clinical setting, demonstrating durable clinical activity among different cancer types. Concerning CC, durable clinical activities were observed especially in the PD-L1 negative population both in chemo naïve and previously pretreated R/M CC population.

Besides, the evidence of an immunomodulatory effect of anti-angiogenetic agents represented an attractive strategy to complement ICIs. Specifically, the aberrant nature of the cancer vessels, along with the production of pro-angiogenic factors by cancer cells, demonstrated to induce both physical and chemical barriers to the immune response, by inhibiting lymphocyte trafficking across endothelia and migration into tumor deposits due to the increase of interstitial pressure, and by the recruitment of immunosuppressive cells due to increased hypoxia within the TME [40–42]. On the other hand, the enhanced expression of coinhibitory factors of the immune response might contribute to anti-angiogenic drugs resistance [23, 43, 44]. Given this strong interplay between the two pathways, the combination of immune checkpoint blockade and tyrosine kinase inhibitors (TKI) targeting the VEGF signal pathway entered the clinic and has been evaluated as a treatment strategy to enhance responses to immunotherapy.

#### Dual Checkpoint Inhibition

The addition of dual checkpoint inhibition to standard chemotherapy with or without bevacizumab represents a promising strategy to cover the full spectrum of the R/M CC population, regardless of PD-L1 expression (Fig. 1). In this context, a bispecific antibody targeting PD-1/CTLA-4, cadonilimab (AK104), was tested in an open-label, phase II study, having enrolled patients in three different cohorts: cohorts A-15 and A-10 were the two cohorts investigating AK104 in combination with platinum-based chemotherapy at two different dosages (AK104 15 mg/kg and 10 mg/kg, respectively) and cohort B-10 was the third cohort investigating AK104 10 mg/kg in combination with platinum-based chemotherapy plus bevacizumab. Impressive results were shown, regardless of PD-L1 status. The secondary endpoint ORR was promising across all three cohorts: specifically, ORRs were 73.3%, 68.8%, and 92.3% in the A-10, A1-15, and B-10 cohorts respectively (Table 3) [45], thus warranting further investigation to evaluate the efficacy in a phase III ongoing trial (NCT04982237, Table 2). Concerning the safety profile, this combination was well tolerated, with the most common grade ≥ 3 treatment-related AEs (TRAEs) belonging to hematological toxicity (anemia: 15.6%, white blood cell count decreased: 11.1%, neutrophil count decreased: 8.9% and platelet count decreased: 8.9%), and grade ≥ 3 immune-related AEs (irAEs) occurring in 17.8% patients. Nonetheless, long-term safety follow-up is awaited.

Besides, the impressive effectiveness of immune checkpoint blockade, which has broken new ground in CC research, further prompts the investigation of immunotherapy in completely replacing chemotherapy as the upfront line of treatment for R/M CC patients (Fig. 1). The phase I/II CheckMate 358 study investigated nivolumab alone or in combination with ipilimumab as a chemotherapy-free regimen for the first-line treatment, showing manageable toxicity and durable tumor regression regardless of tumor PD-L1 expression in patients with R/M CC. With a minimum follow-up of 24 months, the primary endpoint ORR was encouraging either with nivolumab monotherapy (anti PD-1, ORR: 26%, 95% CI 9 to 51%) or in combination with the anti-CTLA-4 inhibitor, ipilimumab, given in two different regimens, namely nivolumab 3 mg/kg every 2 weeks + ipilimumab 1 mg/kg every 6 weeks (N3 + I1 arm, ORR: 39%, 95% CI 17 to 64%), nivolumab 1 mg/kg every 2 weeks + ipilimumab 3 mg/kg every three weeks × 4 cycles followed by nivolumab 240 mg every 2 weeks (N1 + I3 arm, ORR: 41%, 95% CI 29 to 53%), (Table 3). Durable responses were observed across all three arms, with durable responses observed even in the PD-L1 negative population. Notably, higher grade ⩾3 TRAEs (16% versus 5%) and discontinuation rates (24% versus 18%) were observed in arm N1 + I3 arm compared with arm N3 + I1 arm [46].

In addition to the promising role of dual checkpoint inhibition to extend the use of immunotherapy even in the PD-L1 negative, chemo naïve, CC population, this therapeutic strategy was also tested in previously treated R/M CC patients with the aim of overcoming anti-PD-1 resistance (Fig. 1). In the I/II CheckMate-358, the combination of nivolumab (anti-PD-1) and ipilimumab (anti-CTLA-4) was also tested in R/M CC after the failure of the first-line treatment, and regardless of PD-L1 status. Encouraging clinical activity (ORR: 26%, 95% CI 11 to 46 in the N3 + I1 arm; ORR: 35%, 95% CI 21 to 51 in the N1 + I3 arm), along with durable responses (mDoR: 21.1 months, 95% CI, 7.5 to NR in the N3 + I1 arm; mDoR: NR, 95% CI, 5.2 to NR in the N1 + I3 arm at a minimum follow-up of 24 months) were observed.

Similar results were found in a phase II study investigating the safety and efficacy of bastilimab (anti-PD-1) and zalifrelimab (anti-CTLA-4) combination therapy as second-line treatment, with reported ORR of 25.6% (95% CI, 18.8 to 33.9) and mDoR NR (95% CI, 9.7 to NR) after 21 months as a median follow-up (Table 1). The 12-month PFS and OS rates were 21.3% (95% CI, 14.1 to 29.4) and 53.3% (95% CI, 43.8 to 61.9), respectively, with a median PFS of 2.7 months (95% CI, 1.5 to 3.7), and a median OS of 12.8 months (95% CI, 8.8 to 17.6) [47]. Furthermore, this combination strategy was well tolerated, without any unexpected TREAs and with a rate of grade ≥ 3 TRAEs (20%) consistent with previous studies investigating the anti-PD-1/ anti-CTLA-4 combination strategy [47].

Accordingly, the randomized phase RaPiDS/GOG-3028 II study is further assessing the safety and efficacy of bastilimab, both alone and in combination with zalifrelimab, compared with standard chemotherapy in the second-line setting (NCT03894215, Table 2). Besides, promising clinical activity was further observed in the phase II clinical study, with the bispecific antibody AK104, targeting both PD-1 and CTLA-4. Observed ORR was 33% (95% CI, 23.9 to 43.1), and mDoR NR (95% CI, 0.95 to 16.43) at a median follow-up of 9.63 months (Table 1). In line with the previously mentioned trial, tumor response was not related to the PD-L1 status [48]. Moreover, based on results from the first part of a phase II study (CTR20202017) conducted in China, the combination of IBI310 (anti-CTLA-4) and sintilimab (anti-PD-1) received the Breakthrough Therapy Designation by the Center for Drug Evaluation of China’s National Medical Products Administration (NMPA). The combination is under study in phase II, in a randomized trial to compare the efficacy of sintilimab monotherapy to IBI310 (anti-CTLA-4) and sintilimab combination therapy (NCT04590599, Table 2).

Beyond CTLA-4 and PD-1, TIGIT is one of the other checkpoint inhibitory receptors, involved in limiting effector T cell survival and function. TIGIT is structurally and functionally different from CTLA-4 and PD-1, due to its ITIM domain, and because of the ability to further suppress the innate immune response [49]. Several trials are ongoing to investigate the efficacy of PD-1 and TIGIT dual inhibition (NCT04693234, NCT05007106, NCT04300647, Table 2). Preliminary data came from the phase I KEYVIBE-001 study, investigating the pembrolizumab (anti-PD-1)/vibostolimab (anti-TIGIT) combination therapy. In this study pembrolizumab was tested in combination with two different dosages of vibostolimab (200 mg versus 700 mg every3 weeks), demonstrating comparable antitumor activity and safety. After a median follow-up of 11.5 months, ORR of 15% and 23% were observed in the two cohorts (vibostolimab 200 mg versus 700 mg, respectively) and mDoR was not reached (NR, range 10 to 31 + months in the vibostolimab 200 mg cohort; NR, range 4 + to 35 + months in the vibostolimab 700 mg cohort), irrespective to PD-L1 status (Table 1), with evidence of grade ≥ 3 TRAEs in 18%-29% of patients [50]. The recommended phase 2 dose for vibostolimab (200 mg every 3 weeks) is currently under evaluation in combination with pembrolizumab in the phase II KEYVIBE-005 study to evaluate the efficacy both in PD-L1–positive (CPS ≥ 1) and PD-L1–negative CC patients (NCT05007106, Table 2).

#### ICIs in combination with TKI

The first reported study to evaluate the concurrent inhibition of immune checkpoint and angiogenetic pathways in previously treated R/M CC patients was the CLAP trial. In this single-arm, phase II study, the combination of the PD-1 inhibitor camrelizumab with the selectively VEGFR2 inhibitor apatinib demonstrated its clinical activity regardless of the expression of PD-L1. ORR was 55.6% (95% CI, 40.0% to 70.4%), mPFS 8.8 months (95% CI, 5.6 to NR) and after a median follow-up of 11.3 months, neither mDoR nor mOS were reached (mDoR: NR, 95% CI: 5.6 to NR; mOS: NR, 95% CI: 11.6 to NR) [51] (Table 1). Interestingly, an exploratory analysis of the trial aiming at identifying alternative biomarkers to PD-L1 to predict response found a correlation between favorable clinical outcomes and gene alterations in the PI3K/AKT pathway [52]. Based on those encouraging results, camrelizumab-apatinib is currently under study even in the first-line setting, in a randomized, phase 2 trial to assess the efficacy of this combination compared to platinum-based chemotherapy plus bevacizumab in patients with R/M CC patients (NCT04974944, Table 2).

Camrelizumab was further tested in combination with famitinib, a multikinase inhibitor with a broad spectrum of targets, including VEGFR2 and 3, platelet-derived growth factor receptor β (PDGFRβ), FMS-like tyrosine kinase-1/3 receptor, proto-oncogene tyrosine-protein kinase receptor, and the stem-cell factor receptor (Table 1). Efficacy analysis of the phase II single-arm trial showed ORR of 39.4% (95% CI, 22.9 to 57.9), and mPFS of 10.3 months (95% CI, 3.5 to NR). After a median follow-up lasting for 13.6 months, mDoR was NR (95% CI, 8.2 to NR), and the 12-month OS rate was 77.7% (95% CI: 58.9–88.7) [53]. Given this promising clinical activity, along with a manageable safety profile, a randomized, open-label, 3-arm phase 2 trial was developed and is currently ongoing to investigate the efficacy of this combination compared to standard chemotherapy (NCT04680988) in the same setting (Table 2).

Positive signals with the use of TKI to extend ICIs indication to a broad population, further came from the preliminary results of the ENGOT-GYN3/AGO/LIO study. In this trial, PD-1 inhibitor nivolumab was given in combination with lucitanib, a potent inhibitor of VEGFR1–3, PDGFRα/β, and fibroblast growth factor receptor (FGFR) 1–3. Data from stage 1 of the phase II CC cohort were recently reported, showing target lesion reductions even in CC in the PD-L1 negative population, with manageable toxicity (Table 1) [54].

Finally, the FDA-approved combination for endometrial cancer, pembrolizumab, and TKI lenvatinib, is being evaluated in a single-arm phase II trial and the primary endpoint analysis is estimated to be completed by July 2023 (NCT04865887, Table 2).

In contrast to the above mentioned combination strategies tested in the overall previously treated R/M cervical cancer population regardless of PD-L1 status, the anti-PD-1 sintilimab was tested in combination with the multikinase inhibitor anlotinib in a phase II trial, demonstrating its safety and efficacy as second-line or later therapy just for R/M CC patients expressing PD-L1 (Table 1). Interestingly, an exploratory analysis, evaluating other prognostic and predictive biomarkers beyond PD-L1, showed a significant correlation between altered PI3K/AKT signaling and KMT2D with the response, and a negative association of STK11 and/or JAK2 with PFS [55].

### Novel Immunotherapeutic Strategies to Complement or Replace ICIs

The immuno-oncology is rapidly evolving, and a wide range of anticancer drugs with different mechanisms of action are under investigation beyond the immune checkpoint blockade and ADCs. Specifically, the current pipeline in the CC field mainly includes the investigation of adaptive cell therapy (ACT) and the development of cancer vaccines targeting HPV. Those new therapeutic approaches are designed to enhance the immune anticancer response, thus underscoring the urgent need of a deeper knowledge of the TME and its impact on therapeutic response.

#### Tumor-Infiltrating Lymphocytes

A novel immunotherapeutic approach that recently entered the clinic is the use of ACT. Three distinct ACT techniques were developed, with the common aim to stimulate the T cell response within the TME. Non-engineered tumor-infiltrating lymphocytes (TILs) were the first method studied. It consists of T cell extraction directly from the tumor site, ex vivo expansion, activation, and finally re-transfection in the patients [56]. LN-145 is a ready-to-use TILs therapy, which requires the previous administration of a non-myeloablative lymphocyte-depleting preparative regimen before the cell transfer, and the following infusion of interleukin-2 (IL-2). This autologous ACT approach showed promising results, both in the first line and in the subsequent settings (Fig. 1).

In the upfront setting, its efficacy was tested in combination with ICIs, showing striking results in terms of efficacy, and thus potentially representing the best-in-class immunotherapy-based combination strategy. LN-145 in addition to pembrolizumab demonstrated indeed a safety profile and antitumor activity with a reported ORR of 57% (95% CI, 28.9, 82.3) from the C-145–04/innovaTIL-04 trial, enrolling a cohort of chemo naïve CC patients (Table 3) [57].

LN-145 yielded further impressive results also as a single agent in the cohort of R/M CC patients who progressed on or after the upfront treatment. Preliminary results after a median of 3.5 months showed an ORR of 44%, with 11 out of the 12 responders still maintaining their responses at the time of the analysis (Table 1) [58].

Beyond the simple isolation of TILs from tumor samples, a technique to improve the specific recognition of T cells has further developed, through the genetic engineering of T cell receptor (TCR), which is highly specific for the recognition of MHC-restricted peptides [59]. This approach has been deeply studied in the HPV-associated CC subpopulation by several trials investigating the role of different MHC-restricted epitopes of E6/E7 oncoproteins. The first-in-human phase I/II study investigating the T cell therapy rescripted to an epitope of E6 did not show any tumor responses among the 6 patients enrolled in the trial [60]. Yet, a phase II trial generating T cells with HPV-oncoprotein reactivity showed an ORR of 28%, and, more interestingly, prolonged tumor responses were observed regardless of tumor histology (Table 1) [61]. A clinical trial with a higher avidity TCR, targeting the E7 oncoprotein is ongoing to further explore this approach (NCT02858310, Table 2).

Moving forward, the more recent TILs-based approach that entered the clinical setting was the chimeric antigen receptor (CAR) T cell therapy. In this case, T cells are engineered to recognize the tumor-cell surface antigens in an MHC-independent manner, thus overcoming one of the most important mechanisms of tumor immune escape, represented by the downregulation of MHC on cancer cell surfaces [59]. Clinical trials are now ongoing to investigate the applicability of this new treatment strategy even in the CC field, targeting different antigens, including CD22, GD2, PSMA, Muc1, or mesothelin (NCT04556669, NCT03356795, Table 2).

### HPV-Related CC Population: Therapeutic Vaccines and the Transforming Growth Factor-Beta (tgf-β) Signaling Pathway

Other therapeutic approaches were developed for the HPV-related subpopulation, targeting pathways and antigens used by HPV to evade immune surveillance and promote tumor growth. E6 and E7 are the two major oncoproteins mainly involved in driving the CC cells toward oncogenesis, by orchestrating all the hallmarks of cancers, including uncontrolled replication, tumor angiogenesis, invasion, and progression, through the unrestricted telomerase activity, along with the suppression of apoptosis [62]. Thus, therapeutic vaccines targeting E6/E7 oncogenes have represented an attractive tailored strategy for HPV-related cancers to enhance immune response driven by the concurrent blockade of the PD-1/PDL-1 axis. Several trials are currently investigating this combination strategy and preliminary safety and efficacy data were recently reported showing conflicting results. Positive results were observed in phase I/II trial investigating pembrolizumab in combination with GX-188E, a DNA vaccine encoding an E6/E7 fusion protein of HPV16-18 and linked with the Fms-like tyrosine kinase-3 ligand (FLT3L), with increasing antitumor response by stimulating the proliferation of hematopoietic progenitor cells. Significant activity was observed regardless of PD-L1 status, with observed ORR of 31.7% and 25% for the overall HPV16-18 population and the PD-L1-positive subgroup (Table 1) [63]. Accordingly, similar results were achieved in a heavily pretreated, HPV-16 positive, CC population with VB10.16, another therapeutic DNA vaccine encoding the E6/7 fusion protein of HPV16 and linked to the CCL3L1 chemokine via a dimerization module. In a pre-planned interim analysis of a phase II study evaluating the combination of atezolizumab and VB10.16 an overall ORR of 21% was observed, with encouraging clinical activity irrespective of PD-L1 status (ORR of 27% and 17% in the PD-L1-positive and negative population, respectively) [64]. In contrast, the DNA vaccine containing plasmids for E6 and E7 oncogenes for HPV-16/18 and interleukin-12 (IL-12) adjuvant, given in combination with durvalumab failed to demonstrate clinical activity. In the phase II trial evaluating this combination therapy in HVP16/18 related cancers, only one out of twelve patients enrolled in the CC cohort achieves a partial response, thus leading to the study discontinuation for futility. Notably, despite the low ORR, a clinically meaningful disease control rate was observed, and correlative studies are ongoing to further characterize the subgroup of CC patients with prolonged disease control without clinical responses (Table 1) [65]. Overall, DNA vaccines associated with ICIs were well tolerated, with a low incidence of grade ≥ 3 TRAEs (4% with GX-188E [63] and 23% with MEDI0457 [64]) and without any new treatment-emergent AEs compared to prior reports of these agents individually. AESI related to MEDI0457 were observed, including localized reaction and extremity pain at the injection site, none of these grade ≥ 3. The single-arm, phase II ongoing trial evaluating cemiplimab in combination with ISA101b is awaited to clarify the effectiveness of this combination approach (NCT04646005, Table 2). However, it must be pointed out that ISA101b is a structurally different vaccine because of its peptide-based nature. It is composed of long synthetic peptides from the E6/E7 HPV16 that are engineered to be delivered with adjuvant agents to stimulate the adaptive immune system, thus probably inducing a less potent immune response compared to therapeutic DNA vaccines.

Another attractive approach under evaluation in the HPV-selected CC population is the co-targeting of the PD-1/PD-L1 axis and the TGF-β pathway. The first-in-class bifunctional fusion protein Bintrafusp-alfa (M7824) is a new therapeutic drug consisting of two parts: the extracellular domain of TGF-β receptor II and an anti-PD-L1, linked by a flexible linker [66]. Data from phase 1 and phase 2 studies investigating its safety and efficacy in a pretreated, ICIs–naive, R/M CC population are encouraging, showing ORR of 28.2%, mDoR of 11.7 months and mOS of 13.4 months (95% CI, 5.5 to NR; Table 1). Also, toxicity was manageable, without any new safety signal different from previous reports for anti-PDL1 agents and TRAEs known to be related to TGF-β inhibitors (Grade ≥ 3 TRAEs observed: skin lesions, colitis, asymptomatic lipase increase, and gastroparesis with hypokalemia)[67].

## Discussion

Collectively, these data underscore the rapidly evolving landscape of R/M CC management. Until 2018, the only valid therapeutic innovation for these patients was the introduction of bevacizumab in combination with platinum-based chemotherapy in the upfront setting, albeit with the still dismal survival rates. Besides, after the failure of the first-line treatment, no effective therapeutic options were available, thus considering this setting mostly palliative.

The introduction of immunotherapy in the CC treatment armamentarium opened up a new scenario for R/M CC patients, demonstrating improvements in terms of response and survival rates both in the first line and in the subsequent lines of treatment. However, this new opportunity has raised new issues that we still to address. Firstly, the incorporation of ICIs in the first-line setting raised the question of how to manage the post-immunotherapy setting, after the acquired resistance to ICIs. In this context, translational studies are necessary to go through the different pathways that might lead to secondary tumor escape. Besides, a deep investigation of the TME is warranted to understand the complex interactions among the different cellular and molecular components, with the final goal of targeting the immunosuppressive signaling and on the other hand enhancing the antitumor immune response. In this context, the usage of immunomodulatory agents in addition to ICIs, and the concurrent inhibition of different and complementary co-inhibitory signaling pathways may be promising methods to overcome acquired resistance. Secondly, the restriction of immunotherapy to the PD-L1 positive tumors in combination with platinum-based chemotherapy in first-line prompt the investigation of new therapeutic strategies to broaden the use of immunotherapy in PD-L1 negative tumors. In this setting, the immunotherapy combinations might represent an option to explore. Both the dual checkpoint inhibition, and the addition of TILs to ICIs demonstrated to be promising strategies to improve the immunogenicity within the CC patient tumor tissues, regardless of PD-L1 status. Besides, PD-L1 expression might not be the only predictive biomarker for all the immunotherapeutic agents studied in the different trials, and novel molecular signatures to guide the treatment choice are urgently awaited.

Moving forward, the success of ADCs in the CC treatment paved the way for the investigation of tailored therapies targeting cancer-specific antigens and pathways even in the CC field. Several trials are ongoing to investigate the role of novel therapeutic strategies targeting tailored antigens/pathways for the treatment of CC patients; however, more insights into the TME components and cross-talks, as well as into the genomic, transcriptomic profiles of CC patients and their evolving signatures across the different lines of therapies are urgently required to guide treatment choice and to allow the development of a biomarker-selected therapeutic strategy.

## Concluding Remarks

In these years we are undoubtedly witnessing a paradigm shift in the management of R/M CC treatment with rapidly evolving survival rates profoundly improved compared to what we observed just a few years ago. However, more efforts to study mechanisms of resistance and to identify predictive biomarkers are urgently needed, even to establish the optimal sequential strategy for the treatment of this hard-to-treat cancer population. Yet, we have to keep in mind that the majority of CC are preventable, and thus it is even more important to provide access and fill the gap in the cancer screening programs.

## References

[1] Siegel RL, Miller KD, Wagle NS, Jemal A (2023). Cancer statistics, 2023. CA Cancer J Clin.

[2] Cohen CM, Wentzensen N, Castle PE, Schiffman M, Zuna R, Arend RC (2023). Racial and Ethnic Disparities in Cervical Cancer Incidence, Survival, and Mortality by Histologic Subtype. J Clin Oncol.

[3] Singh D, Vignat J, Lorenzoni V, Eslahi M, Ginsburg O, Lauby-Secretan B (2023). Global estimates of incidence and mortality of cervical cancer in 2020: a baseline analysis of the WHO Global Cervical Cancer Elimination Initiative. Lancet Glob Health.

[4] Cibula D, Pötter R, Planchamp F, Avall-Lundqvist E, Fischerova D, Haie Meder C (2018). The European Society of Gynaecological Oncology/European Society for Radiotherapy and Oncology/European Society of Pathology Guidelines for the Management of Patients With Cervical Cancer. Int J Gynecol Cancer.

[5] Chemoradiotherapy for Cervical Cancer Meta-analysis Collaboration (CCCMAC) (2010). Reducing uncertainties about the effects of chemoradiotherapy for cervical cancer: individual patient data meta-analysis. Cochrane Database Syst Rev.

[6] Heinhuis KM, Ros W, Kok M, Steeghs N, Beijnen JH, Schellens JHM (2019). Enhancing antitumor response by combining immune checkpoint inhibitors with chemotherapy in solid tumors. Ann Oncol.

[7] Smola S, Trimble C, Stern PL (2017). Human papillomavirus-driven immune deviation: challenge and novel opportunity for immunotherapy. Ther Adv Vaccines.

[8] Allouch S, Malki A, Allouch A, Gupta I, Vranic S, Al Moustafa AE (2020). High-Risk HPV Oncoproteins and PD-1/PD-L1 Interplay in Human Cervical Cancer: Recent Evidence and Future Directions. Front Oncol.

[9] Otter SJ, Chatterjee J, Stewart AJ, Michael A (2019). The role of biomarkers for the prediction of response to checkpoint immunotherapy and the rationale for the use of checkpoint immunotherapy in cervical cancer. Clin Oncol (R Coll Radiol).

[10] De Felice F, Giudice E, Bolomini G (2021). Pembrolizumab for advanced cervical cancer: safety and efficacy. Expert Rev Anticancer Ther.

[11] Monk BJ, Sill MW, McMeekin DS, Cohn DE, Ramondetta LM, Boardman CH, Benda J, Cella D (2009). Phase III trial of four cisplatin-containing doublet combinations in stage IVB, recurrent, or persistent cervical carcinoma: a Gynecologic Oncology Group study. J Clin Oncol.

[12] Tang X, Zhang Q, Nishitani J, Brown J, Shi S, Le AD (2007). Overexpression of human papillomavirus type 16 oncoproteins enhances hypoxia-inducible factor 1 alpha protein accumulation and vascular endothelial growth factor expression in human cervical carcinoma cells. Clin Cancer Res.

[13] Tewari KS, Sill MW, Long HJ 3rd, et al. Improved survival with bevacizumab in advanced cervical cancer [published correction appears in N Engl J Med. 2017 Aug 17;377(7):702]. N Engl J Med 2014; 370(8):734–743 10.1056/NEJMoa1309748. 10.1056/NEJMoa1309748 PMC4010094 24552320

[14] Tewari KS, Sill MW, Penson RT, Huang H, Ramondetta LM, Landrum LM, Oaknin A, Reid TJ, Leitao MM, Michael HE, DiSaia PJ, Copeland LJ, Creasman WT, Stehman FB, Brady MF, Burger RA, Thigpen JT, Birrer MJ, Waggoner SE, Moore DH, Look KY, Koh WJ, Monk BJ (2017). Bevacizumab for advanced cervical cancer: final overall survival and adverse event analysis of a randomised, controlled, open-label, phase 3 trial (Gynecologic Oncology Group 240). Lancet.

[15] Yu S, Garcia AA (2015). Advancements in recurrent and metastatic cervical cancer. Am J Hematol-Oncol.

[16] Frenel JS, Le Tourneau C, O’Neil B (2017). Safety and efficacy of pembrolizumab in advanced, programmed death ligand 1-positive cervical cancer: results from the phase Ib KEYNOTE-028 Trial. J Clin Oncol.

[17] •• Chung HC, Delord JP, Peters R, et al. Efficacy and safety of pembrolizumab treatment of advanced cervical cancer: updated results from the phase II KEYNOTE-158 study. Gynecologic Oncol 20201;162:S27. 10.1016/S0090-8258(21)00696-X. **This study led to the FDA approval of pembrolizumab as second-line treatment for CC patients with combined CPS≥1.**

[18] Wu X, Xia L, Wang J (2022). Efficacy and safety of zimberelimab (GLS-010) monotherapy in patients with recurrent or metastatic cervical cancer: a multicenter, open-label, single-arm, phase II study. Annal Oncol.

[19] Santin AD, Deng W, Frumovitz M (2020). Phase II evaluation of nivolumab in the treatment of persistent or recurrent cervical cancer (NCT02257528/NRG-GY002). Gynecol Oncol.

[20] O’Malley DM, Oaknin A, Monk BJ (2021). Phase II study of the safety and efficacy of the anti-PD-1 antibody balstilimab in patients with recurrent and/or metastatic cervical cancer. Gynecol Oncol.

[21] Lheureux S, Butler MO, Clarke B (2018). association of ipilimumab with safety and antitumor activity in women with metastatic or recurrent human papillomavirus-related cervical carcinoma. JAMA Oncol.

[22] Tewari KS, Monk BJ, Vergote I (2022). Survival with cemiplimab in recurrent cervical cancer. N Engl J Med.

[23] Khan KA, Kerbel RS (2018). Improving immunotherapy outcomes with anti-angiogenic treatments and vice versa. Nat Rev Clin Oncol.

[24] •• Colombo N, Dubot C, Lorusso D, et al. Pembrolizumab for persistent, recurrent, or metastatic cervical cancer. N Engl J Med. 2021;385(20):1856–1867. **This study led to the FDA and EMA approval of pembrolizumab in combination with platinum-based chemotherapy** ± (**bevacizumab as first-line treatment for CC patients with CPS ≥1.**) 10.1056/NEJMoa2112435 34534429

[25] Grau JF, Farinas-Madrid L, Oaknin A (2020). A randomized phase III trial of platinum chemotherapy plus paclitaxel with bevacizumab and atezolizumab versus platinum chemotherapy plus paclitaxel and bevacizumab in metastatic (stage IVB), persistent, or recurrent carcinoma of the cervix: the BEATcc study (ENGOT-Cx10/GEICO 68-C/JGOG1084/GOG-3030). Int J Gynecol Cancer.

[26] Liu J, Fang C, Zhou Q (2022). A phase II, open-label, single-arm study of QL1604 plus paclitaxel-cisplatin/carboplatin as first-line treatment in patients with recurrent or metastatic cervical cancer. Annal Oncol.

[27] Fu Z, Li S, Han S, Shi C, Zhang Y (2022). Antibody drug conjugate: the “biological missile” for targeted cancer therapy. Signal Transduct Target Ther.

[28] Liu Y, Jiang P, Capkova K (2011). Tissue factor-activated coagulation cascade in the tumor microenvironment is critical for tumor progression and an effective target for therapy. Cancer Res.

[29] Breij EC, de Goeij BE, Verploegen S (2014). An antibody-drug conjugate that targets tissue factor exhibits potent therapeutic activity against a broad range of solid tumors. Cancer Res.

[30] de Bono JS, Concin N, Hong DS (2019). Tisotumab vedotin in patients with advanced or metastatic solid tumours (InnovaTV 201): a first-in-human, multicentre, phase 1–2 trial. Lancet Oncol.

[31] Hong DS, Concin N, Vergote I (2020). Tisotumab Vedotin in Previously Treated Recurrent or Metastatic Cervical Cancer. Clin Cancer Res.

[32] Coleman RL, Lorusso D, Gennigens C (2021). Efficacy and safety of tisotumab vedotin in previously treated recurrent or metastatic cervical cancer (innovaTV 204/GOG-3023/ENGOT-cx6): a multicentre, open-label, single-arm, phase 2 study. Lancet Oncol.

[33] Yonemori K, Kuboki Y, Hasegawa K (2022). Tisotumab vedotin in Japanese patients with recurrent/metastatic cervical cancer: Results from the innovaTV 206 study. Cancer Sci.

[34] Vergote I, Monk BJ, O’Cearbhaill RE (2021). Tisotumab vedotin (TV) + carboplatin (Carbo) in first-line (1L) or + pembrolizumab (Pembro) in previously treated (2L/3L) recurrent or metastatic cervical cancer (r/mCC): Interim results of ENGOT-Cx8/GOG-3024/innovaTV 205 study. Annal Oncol.

[35] Müller P, Martin K, Theurich S (2014). Microtubule-depolymerizing agents used in antibody-drug conjugates induce antitumor immunity by stimulation of dendritic cells. Cancer Immunol Res.

[36] Rios-Doria J, Harper J, Rothstein R (2017). Antibody-drug conjugates bearing pyrrolobenzodiazepine or tubulysin payloads are immunomodulatory and synergize with multiple immunotherapies. Cancer Res.

[37] Lorusso D, Vergote I, O’Cearbhaill RE, et al. Tisotumab vedotin (TV) + pembrolizumab (pembro) in first-line (1L) recurrent or metastatic cervical cancer (r/mCC): Interim results of ENGOT Cx8/GOG 3024/innovaTV 205. Journal of Clinical Oncology 2022 40:16_suppl, 5507–5507.

[38] Vergote I, Mirza MR, Sehouli J, et al., Trial in progress update on ENGOT-cx8/GOG-3024/innovaTV 205: Addition of a new cohort with first-line (1L) tisotumab vedotin (TV) + pembrolizumab (pembro) + carboplatin (carbo) ± bevacizumab (bev) in recurrent/metastatic cervical cancer (r/mCC). Journal of Clinical Oncology 2022 40:16_suppl, TPS5603-TPS5603.

[39] Fife BT, Bluestone JA (2008). Control of peripheral T-cell tolerance and autoimmunity via the CTLA-4 and PD-1 pathways. Immunol Rev.

[40] Barsoum IB, Smallwood CA, Siemens DR (2014). A mechanism of hypoxia-mediated escape from adaptive immunity in cancer cells. Cancer Res.

[41] Fukumura D, Kloepper J, Amoozgar Z, Duda DG, Jain RK (2018). Enhancing cancer immunotherapy using antiangiogenics: opportunities and challenges. Nat Rev Clin Oncol.

[42] Oelkrug C, Ramage JM (2014). Enhancement of T cell recruitment and infiltration into tumours. Clin Exp Immunol.

[43] Rahma OE, Hodi FS (2019). The Intersection between Tumor Angiogenesis and Immune Suppression. Clin Cancer Res.

[44] Vitale I, Shema E, Loi S, Galluzzi L (2021). Intratumoral heterogeneity in cancer progression and response to immunotherapy. Nat Med.

[45] Wang J, Lou H, Cai HB (2022). A study of AK104 (an anti-PD1 and anti-CTLA4 bispecific antibody) combined with standard therapy for the first-line treatment of persistent, recurrent, or metastatic cervical cancer (R/M CC). J Clin Oncol.

[46] Oaknin A, et al. Safety and efficacy of nivolumab (NIVO) ± ipilimumab (IPI) in patients (pts) with recurrent/metastatic cervical cancer (R/M CX CA) in CHECKMATE 358. ESMO Congress 2022, Abstract 520MO.

[47] O’Malley DM, Neffa M, Monk BJ (2022). Dual PD-1 and CTLA-4 checkpoint blockade using balstilimab and zalifrelimab combination as second-line treatment for advanced cervical cancer: an open-label phase II study. J Clin Oncol.

[48] Xiaohua Wu, et al. Efficacy and safety of cadonilimab, an Anti-PD-1/CTLA4 Bi-specific antibody, in previously treated recurrent or metastatic (R/M) cervical cancer: a multicenter, open-label, single-arm, phase II trial. 2022 SGO, abstract #72.

[49] Chauvin JM, Zarour HM (2020). TIGIT in cancer immunotherapy. J Immunother Cancer.

[50] Shapira-Frommer R, Perets R, Voskoboynik, et al. Safety and efficacy of vibostolimab plus pembrolizumab in patients with cervical cancer naive to PD-1/PD-1 inhibitors. Presented at: 2022 AACR Annual Meeting; April 8–13, 2022; New Orleans, LA. Abstract CT508.

[51] Lan C, Shen J, Wang Y (2020). Camrelizumab Plus Apatinib in Patients With Advanced Cervical Cancer (CLAP): A Multicenter, Open-Label, Single-Arm. Phase II Trial J Clin Oncol.

[52] Huang X, He M, Peng H (2021). Genomic profiling of advanced cervical cancer to predict response to programmed death-1 inhibitor combination therapy: a secondary analysis of the CLAP trial. J Immunother Cancer.

[53] Xia L, Zhou Q, Gao Y (2022). A multicenter phase 2 trial of camrelizumab plus famitinib for women with recurrent or metastatic cervical squamous cell carcinoma. Nat Commun.

[54] Patel MR, Makker V, Oaknin A (2022). Efficacy and safety of lucitanib plus nivolumab in patients with advanced gynecologic malignancies: Phase 2 results from the LIO-1 study (NCT04042116; ENGOT-GYN3/AGO/LIO). J Clin Oncol.

[55] Xu Q, Wang J, Sun Y (2022). Efficacy and safety of sintilimab plus anlotinib for PD-L1-positive recurrent or metastatic cervical cancer: a multicenter, single-arm, prospective phase II trial. J Clin Oncol.

[56] Morotti M, Albukhari A, Alsaadi A, Artibani M, Brenton JD, Curbishley SM, Dong T, Dustin ML, Hu Z, McGranahan N, Miller ML, Santana-Gonzalez L, Seymour LW, Shi T, Van Loo P, Yau C, White H, Wietek N, Church DN, Wedge DC, Ahmed AA (2021). Promises and challenges of adoptive T-cell therapies for solid tumours. Br J Cancer.

[57] O’Malley D, Lee SM, Psyrri A, et al. Phase 2 efficacy and safety of autologous tumor-infiltrating lymphocyte (TIL) cell therapy in combination with pembrolizumab in immune checkpoint inhibitor-naïve patients with advanced cancers. Presented at: The Society For immunotherapy of Cancer 36th Annual Meeting; November 2021; Washington, D.C.

[58] Jazaeri AA, Zsiros E, Amaria RN (2019). Safety and efficacy of adoptive cell transfer using autologous tumor infiltrating lymphocytes (LN-145) for treatment of recurrent, metastatic, or persistent cervical carcinoma. J Clin Oncol.

[59] Harris DT, Kranz DM (2013). Adoptive T cell therapies: a comparison of T cell receptors and chimeric antigen receptors. Trends Pharmacol Sci.

[60] Doran SL, Stevanović S, Adhikary S, Gartner JJ, Jia L, Kwong MLM, Faquin WC, Hewitt SM, Sherry RM, Yang JC, Rosenberg SA, Hinrichs CS (2019). T-cell receptor gene therapy for human papillomavirus-associated epithelial cancers: a first-in-human, phase I/II study. J Clin Oncol.

[61] Stevanović S, Helman SR, Wunderlich JR (2019). a phase ii Study of Tumor-infiltrating Lymphocyte Therapy for Human Papillomavirus-associated Epithelial Cancers. Clin Cancer Res.

[62] Pal A, Kundu R (2020). Human papillomavirus E6 and E7: the cervical cancer hallmarks and targets for therapy. Front Microbiol.

[63] Lee S, Lim M-C, Kim YM (2022). Efficacy and safety of GX-188E, a therapeutic DNA vaccine, combined with pembrolizumab in HPV 16- and/or 18-positive advanced cervical cancer (phase II): safe and effective in both PD-L1 positive and negative. Ann Oncol.

[64] Nykode therapeutics announces positive interim results from its phase 2 trial with VB10.16 in combination with immune checkpoint inhibitor atezolizumab in advanced cervical cancer. News release. Nykode Therapeutics. May 9, 2022. Accessed May 10, 2022. https://bit.ly/38cY98o.

[65] Morris VK, Jazaeri AA, Westin SN (2021). Phase II trial of MEDI0457 and durvalumab for patients with recurrent/metastatic HPV-associated cancers. J Clin Oncol.

[66] Strauss J, Gatti-Mays ME, Cho BC (2020). Bintrafusp alfa, a bifunctional fusion protein targeting TGF-β and PD-L1, in patients with human papillomavirus-associated malignancies. J Immunother Cancer.

[67] Strauss J, Fadi S, Braiteh FS, Calvo E (2021). Evaluation of bintrafusp alfa, a bifunctional fusion protein targeting TGF-β and PD-L1, in cervical cancer: data from phase 1 and phase 2 studies. J Clini Oncol.

